# Supplementary material for: Splice-Junction-Based Mapping of Alternative Isoforms in the Human Proteome
Source: Cell Rep. Author manuscript; Available in PMC 2020 Jan 15. (PMC6961840; doi:10.1016/j.celrep.2019.11.026)
Supplement: 2 [file NIHMS1546469-supplement-2.zip › DF1/PXD010154/Liver/Liver_72_RPL35_AVEGALSWESLDR.pdf]

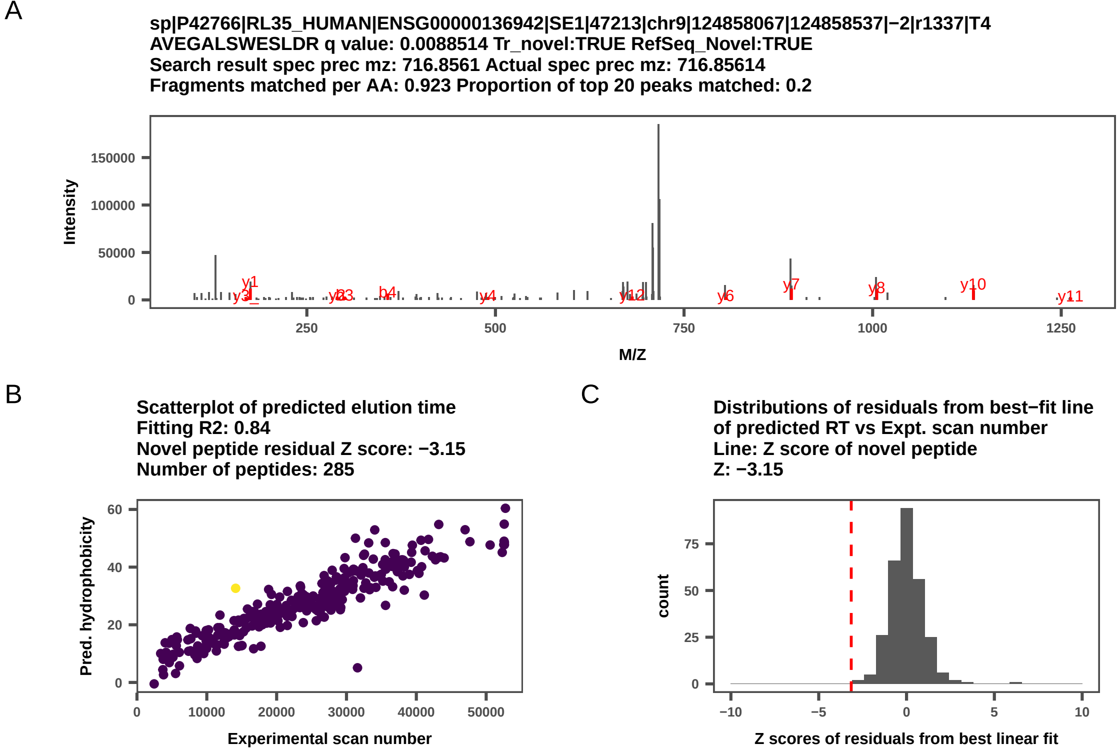
A
sp|P42766|RL35_HUMAN|ENSG00000136942|SE1|47213|chr9|124858067|124858537|−2|r1337|T4
AVEGALSWESLDR q value: 0.0088514 Tr_novel:TRUE RefSeq_Novel:TRUE
Search result spec prec mz: 716.8561 Actual spec prec mz: 716.85614
Fragments matched per AA: 0.923 Proportion of top 20 peaks matched: 0.2
Intensity
150000
100000
50000
0
y1
b4
y4
y6
y7
y8
y10
y11
250
500
750
1000
1250
M/Z
B
Scatterplot of predicted elution time
Fitting R2: 0.84
Novel peptide residual Z score: −3.15
Number of peptides: 285
Pred. hydrophobicity
60
40
20
0
0
10000
20000
30000
40000
50000
Experimental scan number
C
Distributions of residuals from best−fit line
of predicted RT vs Expt. scan number
Line: Z score of novel peptide
Z: −3.15
count
75
50
25
0
−10
−5
0
5
10
Z scores of residuals from best linear fit
